# Meta-synthesis of qualitative research on comfort experiences of family caregivers in hospice care: a systematic review

**DOI:** 10.3389/fpubh.2026.1819762

**Published:** 2026-04-20

**Authors:** Hui Huang, Yanling Cai, Sha Miao, Lei Luo

**Affiliations:** 1 Department of Anesthesiology, Sichuan Clinical Research Center for Cancer, Sichuan Cancer Hospital & Institute, Sichuan Cancer Center, University of Electronic Science and Technology of China, Chengdu, China; 2 Department of Breast Surgery, Sichuan Clinical Research Center for Cancer, Sichuan Cancer Hospital & Institute, Sichuan Cancer Center, University of Electronic Science and Technology of China, Chengdu, China; 3 Department of Abdominal Radiotherapy, Sichuan Clinical Research Center for Cancer, Sichuan Cancer Hospital & Institute, Sichuan Cancer Center, University of Electronic Science and Technology of China, Chengdu, China; 4 General Affairs Office, Sichuan Clinical Research Center for Cancer, Sichuan Cancer Hospital & Institute, Sichuan Cancer Center, University of Electronic Science and Technology of China, Chengdu, China

**Keywords:** caregivers, comfort, hospice care, meta-synthesis, qualitative research

## Abstract

**Objective:**

Globally, only 14% of end-of-life patients receive hospice care. Family caregivers, who bear the long-term caregiving burden, have become “hidden patients,” and their multidimensional comfort experiences encompassing physical, psychological, social, and economic aspects—urgently require attention. However, to date, no qualitative systematic review focusing on the comfort experiences of family caregivers in hospice care has been conducted either domestically or internationally. Therefore, this study employed a meta- synthesis approach to systematically synthesize caregivers’ comfort experiences, aiming to propose strategies to alleviate core issues such as caregiving strain, psychological distress, financial burden, role conflict, and insufficient social support, thereby providing evidence-based guidance for developing hospice care interventions centered on enhancing caregiver comfort.

**Methods:**

A systematic search was conducted in PubMed, Cochrane Library, Web of Science, CNKI, Wanfang, and VIP databases to identify qualitative studies on the comfort experiences of family caregivers in hospice care. The search period extended from database inception to December 2025. Methodological quality of the included studies was assessed using the Joanna Briggs Institute (JBI) Critical Appraisal Checklist for Qualitative Research. A meta-aggregation approach was employed to synthesize the findings.

**Results:**

A total of eight studies were included. From these, 24 findings were extracted, which were subsequently grouped into eight categories, and finally synthesized into three synthesized findings. Synthesized finding 1: Discomfort and changes experienced by caregivers of end-of-life patients. Synthesized finding 2: Facilitating factors that enhance the comfort of family caregivers during hospice care. Synthesized finding 3: Family caregivers’ pursuit of multifaceted support.

**Conclusion:**

Family caregivers encounter various comfort-related challenges during hospice care. These findings highlight the urgent need to address caregivers’ physical health and spiritual needs, provide professional knowledge and death education, and mobilize governments, healthcare institutions, and non-governmental organizations to offer economic support and institutional safeguards.

## 1 Introduction

The World Health Organization (WHO) states that approximately 56.8 million end-of-life patients globally require hospice care services annually. However, only 14% of these patients receive systematic care (1). There is a severe shortage of care resources for end-of-life patients. Consequently, family caregivers must undertake the primary caregiving responsibilities. During long-term care, caregivers witness the deterioration of their family member’s illness and endure prolonged, high-intensity caregiving pressure. This situation effectively turns caregivers into hidden patients (2–4). Studies indicate that family caregivers in hospice care often experience physical exhaustion and sleep deprivation due to heavy caregiving tasks. When facing the deterioration and death of the patient, they frequently feel sadness, anxiety, and helplessness (5, 6). At the same time, reduced social interactions make them prone to loneliness, and a lack of understanding or support from family or society further exacerbates their emotional distress (7). Moreover, the hospice environment is often tense, lacking privacy and rest spaces, leading to multidimensional discomfort among caregivers (8, 9). For patients, comfort aims to preserve dignity, alleviate symptoms, and improve quality of life at the end of life. For caregivers, comfort helps sustain their caregiving capacity, mental health, and overall well-being. Discomfort among family caregivers—such as caregiving burnout, anxiety, and unresolved grief—can negatively affect patient outcomes, including reduced care quality, increased symptom burden, and weakened emotional support (10, 11).

Hospice care, as an approach to improve the quality of life for both patients and caregivers, primarily enhances comfort through the early identification, assessment, and alleviation of symptoms (12). According to Kolcaba’s Theory of Holistic Comfort, comfort is the core component of hospice care and encompasses four dimensions: physical, psychospiritual, sociocultural, and environmental (13). In the hospice context, caregivers serve both as providers and recipients of care. Their comfort needs include understanding, self-efficacy, meaning-making, formal and informal support, resource access, and self-care (14, 15).

Currently, qualitative research on the needs of family caregivers in hospice care is increasingly conducted both domestically and internationally. However, the findings remain relatively homogeneous (16), and a systematic review that analyzes caregivers’ needs and problems from a multidimensional perspective of comfort experiences is still lacking. Meta-synthesis provides a valuable method for synthesizing qualitative evidence, enabling a deeper exploration of complex emotional and psychological processes (17). It is particularly suitable for capturing the multidimensional emotional trajectories and dynamic processes of caregivers of end-of-life patients.

Therefore, this study will retrieve relevant qualitative literature from both domestic and international sources and conduct a meta-synthesis to: clarify the characteristics and influencing factors of comfort experiences among family caregivers of end-of-life patients throughout the hospice care process; alleviate caregivers’ caregiving burden and psychological stress; and provide evidence-based support for developing hospice care interventions, thereby simultaneously improving the comfort level and quality of life of both patients and their family caregivers.

## 2 Materials and methods

### 2.1 Literature inclusion and exclusion criteria

The inclusion and exclusion criteria were developed according to the PICoS framework (18).

The inclusion criteria were as follows:

(1) Population (P): Family caregivers undertaking the primary caregiving responsibility for patients receiving hospice care. Caregivers were required to be relatives of the patient, aged ≥18 years, and with a caregiving duration of ≥3 months.
(2) Phenomena of Interest (I): The comfort experiences and needs of family caregivers during the caregiving process.
(3) Context (Co): The care process involving family caregivers, either during the patient’s hospitalization or after discharge.
(4) Study Design (S): Qualitative studies, including but not limited to phenomenological, grounded theory, and ethnographic research.

Exclusion criteria were:

(1) Studies involving caregivers with impaired consciousness.
(2) Duplicate publications.
(3) Studies for which the full text could not be accessed.

### 2.2 Literature search strategy

A systematic search was conducted in seven English-language databases (PubMed, Cochrane Library, Web of Science) and three Chinese-language databases (CNKI, Wanfang, and VIP) to identify qualitative studies on the comfort experiences of family caregivers in hospice care. A combination of free-text terms and controlled vocabulary (subject headings) was used. The search timeframe was from database inception to December 2025. Additionally, the reference lists of all included studies were manually screened to identify further relevant publications.

English and Chinese search terms: Hospice care terms: “hospice and palliative care nursing” OR “palliative nursing” OR “palliative care nursing” OR “hospice nursing” OR “hospice care” OR “palliative care” OR “palliative treatment.” Comfort terms: “Patient comfort” OR “comfort care” OR “comfort.” Caregiver terms: “caregiver” OR “spouse caregiver” OR “family caregiver*.” Qualitative research terms: “qualitative method” OR “qualitative research” OR “qualitative study” OR “descriptive study” OR “phenomenology” OR “grounded theory” OR “hermeneutic” OR “thematic analysis.”

### 2.3 Literature screening and data extraction

Literature screening was conducted independently by two reviewers based on the inclusion and exclusion criteria. The screening process involved two stages: first, titles and abstracts were screened to exclude clearly irrelevant records; subsequently, the full texts of potentially eligible studies were independently reviewed by the two reviewers, and consensus was reached on the final inclusion. Any disagreements between the two reviewers were resolved through discussion with a third reviewer.

For data extraction, a standardized data extraction form was pre-designed based on the research questions and the JBI methodological guidelines for qualitative systematic reviews. The form included the following items: publication year, country, research methodology, study participants (caregiver age, gender, relationship to the patient, caregiving duration), research focus, key findings related to comfort experiences (themes, categories, and representative quotations), facilitating factors and barriers influencing comfort, and limitations reported by the authors. Data extraction was performed independently by two reviewers. Each reviewer read the full text and extracted data without discussion during the extraction process. After independent extraction, the two reviewers cross-checked their results. Any discrepancies were resolved through discussion until consensus was reached; if disagreements persisted, a third reviewer made the final decision. When information was unclear or key data were missing, the corresponding author was contacted by email to request clarification or missing data. All extracted data were entered into an Excel spreadsheet (Microsoft Excel 2019) using dual independent entry followed by cross-verification.

### 2.4 Methodological quality assessment

The methodological quality of the included studies was assessed using the Joanna Briggs Institute (JBI) Critical Appraisal Checklist for Qualitative Research (19). The tool comprises 10 items assessing aspects such as philosophical perspective, research question, data collection methods, and consistency between the research methodology and the interpretation of results. Based on this assessment, studies were graded as follows: Grade A (all criteria met, minimal risk of bias), Grade B (some criteria met, moderate risk of bias), and Grade C (no criteria met, high risk of bias). Two reviewers independently assessed the quality of each study. In case of disagreements, a third reviewer was consulted to reach a consensus on eligibility. Subsequently, only studies graded A or B were included, while those graded C were excluded.

### 2.5 Meta-synthesis method

A meta-aggregation approach was used to synthesize the findings of the included studies. Two researchers independently read the included qualitative studies and extracted findings relevant to the research question. The extracted data were entered into a standardized data extraction form. During this process, the two researchers cross-checked each other’s work to ensure the accuracy and consistency of the extracted findings.

Prior to synthesis, two researchers independently graded each extracted finding using the JBI credibility assessment criteria for qualitative research. The findings were classified into three levels: (1) Unequivocal: the finding is supported by clear verbatim participant quotations, and the researchers’ interpretation aligns with the quotation content in a logically coherent and easily understandable manner. (2) Equivocal: the finding is supported by participant quotations, but there is some inconsistency between the researchers’ interpretation and the quotation content, or the quotation itself is ambiguous or lacks sufficient elaboration. (3) Unsupported: the finding lacks clear support from verbatim participant quotations and is based primarily on the researchers’ personal views. After the two researchers independently completed the grading, they cross-checked their results. Any disagreements were resolved through discussion until consensus was reached. If consensus could not be achieved, a third researcher was consulted to make the final determination. Only findings graded as “unequivocal” or “equivocal” were included in the subsequent synthesis, while those graded as “unsupported” were excluded to ensure the reliability of the synthesized findings.

After thoroughly understanding the methodology and philosophical underpinnings of each qualitative study, the findings were iteratively read, analyzed, and interpreted. Similar findings were then synthesized into categories based on their thematic content, with each category containing two or more related findings. Subsequently, the categories were further analyzed to identify relationships and connections among them. Finally, these categories were aggregated into synthesized findings, with each synthesized finding comprising at least one category. The synthesized findings represent a systematic summary and integration of the original findings, consistent with the descriptive nature of the meta-aggregation approach, rather than generating novel interpretations or theoretical constructions beyond the original data.

## 3 Results

### 3.1 Literature screening results

**Figure 1:**
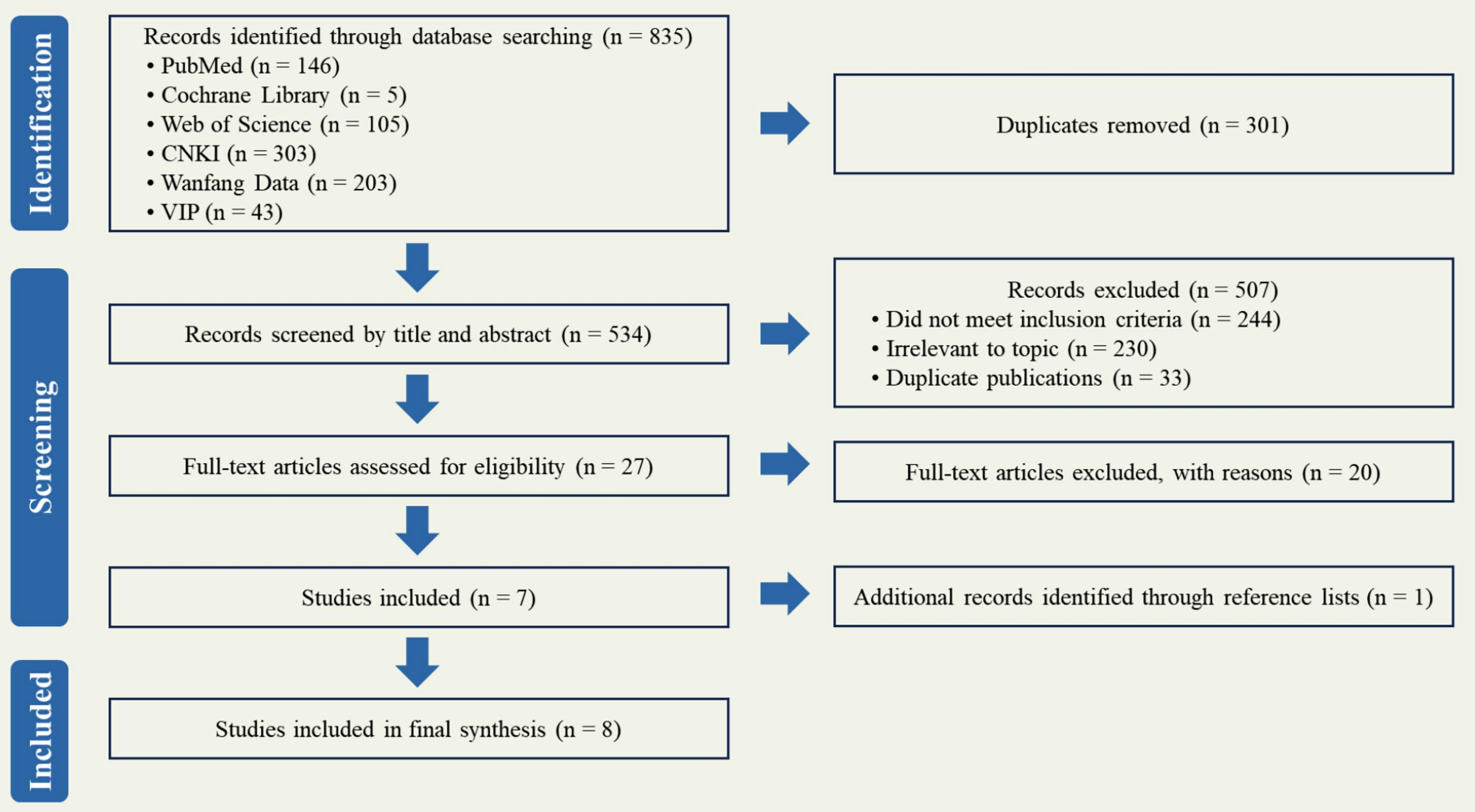
Literature retrieval and screening process.

A systematic search was performed in the designated databases, which yielded a total of 853 records. EndNote (version X9) was used to manage citations and articles throughout the review process. The screening process was conducted as follows: (1) After duplicates were removed using EndNote, 534 records remained. (2) Following the initial screening of titles and abstracts, 27 articles were identified as potentially relevant. (3) Subsequently, the full texts of these 27 articles were reviewed against the inclusion and exclusion criteria. (4) After full-text review, 19 articles that did not meet the criteria were excluded, resulting in a final inclusion of 8 studies. The literature retrieval and screening process are shown in Figure 1.

### 3.2 Characteristics of included studies

A total of eight studies were included, with the majority employing a phenomenological design. The key characteristics of the included studies are presented in Table 1.

**Table 1:** Characteristics of included studies.

Included literature	Time	Country	Research method	Participants	Phenomenon of interest	Main results
Twahirwa et al. (23)	2025	Rwanda	Phenomenological study	20 family caregivers of end-of-life patients	Exploring the actual experiences and care challenges of informal home care providers delivering palliative care	Lack of money and materials Fear and discomfort of witnessing the illness Lack of professional training Social role conflict Structural and Systemic Challenges
Benites et al. (22)	2022	Brazil	Phenomenological study	16 family caregivers of hospitalized patients with advanced cancer	Understanding the care experiences and spiritual needs of family caregivers of patients with advanced cancer in Brazil	Need for family support Complex emotional changes Reduction of invasive measures to enhance patient comfort Emotional neglect
Washington et al. (21)	2021	USA	Phenomenological study	39 family caregivers of patients receiving palliative cancer care	Understanding the comfort needs of caregivers of patients in palliative care	Need for support from friends and family Need for support from healthcare professionals Need for professional healthcare resources to meet basic needs and fulfill caregiving responsibilities Caregivers’ self-care needs
Perao et al. (27)	2021	Brazil	Phenomenological study	30 family members of patients receiving palliative care	Understanding the social representations of comfort among family members of patients in palliative care	Increased negative emotions Communication and interaction with the healthcare team Professional competence of the multidisciplinary team Comfortable care environment
Wu et al. (26)	2019	China	Phenomenological study	10 family caregivers of patients with end-stage cancer receiving hospice care	Exploring the care burden and needs of caregivers of patients with end-stage cancer in hospice care	Disruption of daily life Negative psychological experiences Desire for a professional multidisciplinary hospice care team Desire for care-related knowledge and information
Ferrell et al. (20)	2018	USA	Phenomenological study	20 family caregivers of patients with cancer	Understanding the problems and comfort needs of caregivers of patients with cancer	Sleep insufficiency Emotional sensitivity Social isolation Seeking professional support Spiritual needs and faith-based support
Leow et al. (25)	2017	Singapore	Phenomenological study	19 family caregivers of patients with advanced cancer	Exploring the challenges, emotions, and coping strategies of family caregivers of patients with advanced cancer	Caregiving challenges Negative emotions Coping strategies Positive aspects of caregiving
Jin et al. (24)	2016	China	Phenomenological study	10 spouses of patients with advanced cancer	Understanding the lived experiences of spouses of patients with advanced cancer in home-like wards during the caregiving process	Feelings of complaint and helplessness Feelings of uncertainty and loss Feelings of satisfaction and self-worth Adjusting mindset and accepting reality

### 3.3 Quality assessment results of included studies

The eight included studies were assessed using the Joanna Briggs Institute (JBI) Critical Appraisal Checklist for Qualitative Research (2020). The assessment results indicated that seven studies were rated as Grade B and one as Grade A. The detailed quality assessment results are presented in Table 2.

**Table 2:** Methodological quality evaluation of included literatures.

Included literature	①	②	③	④	⑤	⑥	⑦	⑧	⑨	⑩	Quality grade
Twahirwa et al. (23)	Yes	Yes	Yes	Yes	Yes	Yes	No	Yes	Yes	Yes	B
Benites et al. (22)	Yes	Yes	Yes	Yes	Yes	Yes	Yes	Yes	Yes	Yes	A
Washington et al. (21)	Yes	Yes	Yes	Yes	Yes	Yes	Unclear	Yes	Yes	Yes	B
Perao et al. (27)	Yes	Yes	Yes	Yes	Yes	No	No	Yes	Yes	Yes	B
Wu et al. (26)	Yes	Yes	Yes	Yes	Yes	Unclear	No	Yes	Yes	Yes	B
Ferrell et al. (20)	Yes	Yes	Yes	Yes	Yes	Yes	No	Yes	Yes	Yes	B
Leow et al. (25)	Yes	Yes	Yes	Yes	Yes	No	No	Yes	Yes	Yes	B
Jin et al. (24)	Yes	Yes	Yes	Yes	Yes	No	Yes	Yes	Yes	Yes	B

The JBI Critical Appraisal Checklist for Qualitative Research comprises the following 10 items:

(1) Is there congruity between the stated philosophical perspective and the research methodology?

(2) Is there congruity between the research methodology and the research question or objectives

(3) Is there congruity between the research methodology and the methods used to collect data?

(4) Is there congruity between the research methodology and the representation and analysis of data?

(5) Is there congruity between the research methodology and the interpretation of results?

(6) Is there a statement locating the researcher culturally or theoretically?

(7) Is the influence of the researcher on the research, and vice versa, addressed?

(8) Are participants, and their voices, adequately represented?

(9) Is the research ethical according to current criteria or, for recent studies, is there evidence of ethical approval by an appropriate body?

(10) Do the conclusions draw in the research report flow from the analysis, or interpretation, of the data?

### 3.4 Meta-synthesis results

**Figure 2:**
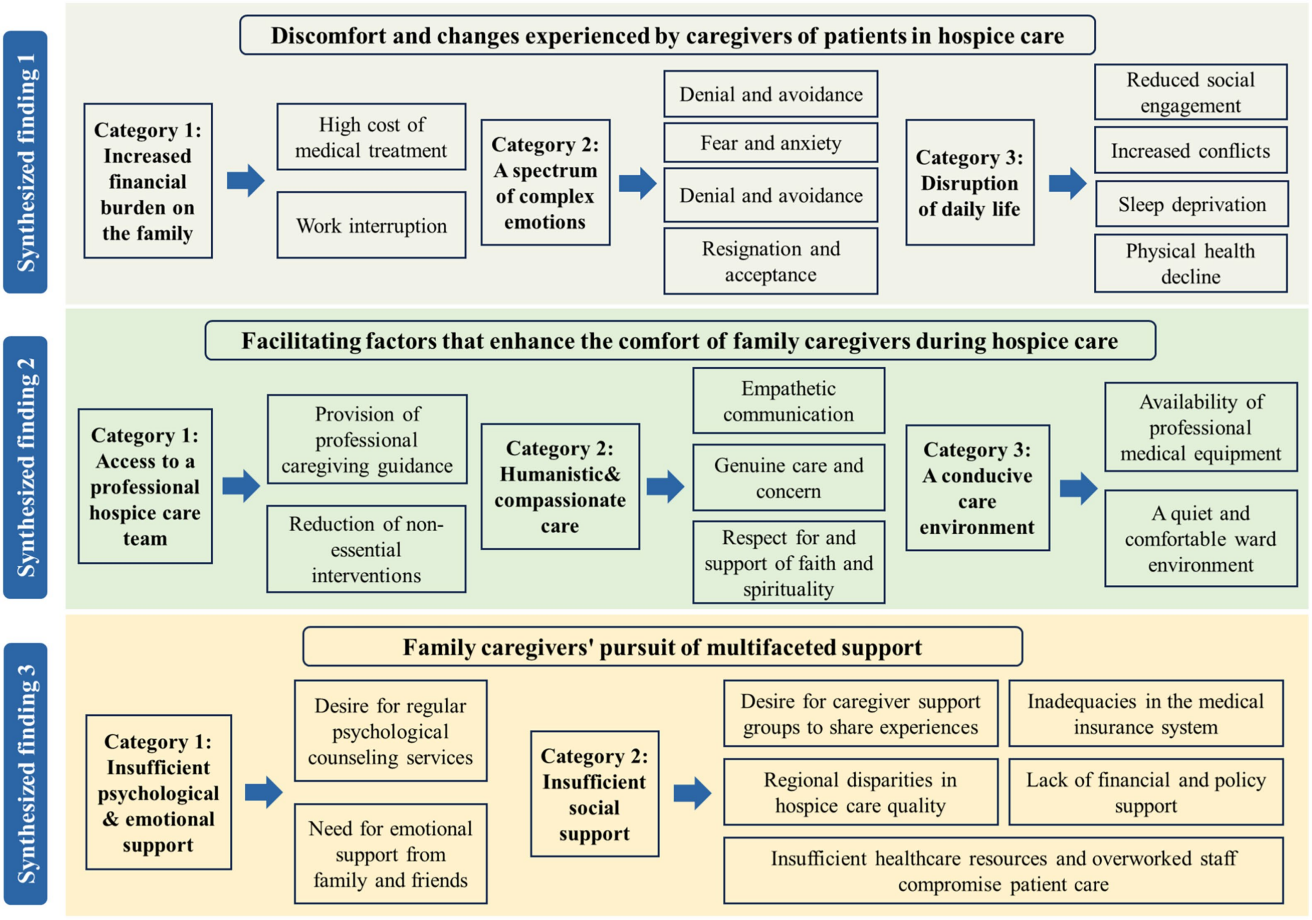
Theme map of synthesized findings on comfort experiences of family caregivers in hospice care.

A total of 24 findings were extracted from the eight included studies. Through iterative reading, analysis, and comparison, similar findings were grouped into eight categories, which were subsequently synthesized into three overarching synthesized findings, as shown in Figure 2. A category is a preliminary grouping of research findings that share similar themes and meanings, reflecting a specific aspect of caregivers’ comfort experiences. A synthesized finding, in contrast, is a further summarization and generalization of the content of categories based on analyzing the intrinsic relationships (e.g., causal, parallel, and progressive) among multiple categories. For example, categories such as “increased family financial burden” and “A spectrum of complex emotions” were integrated into the synthesized finding “discomfort and changes experienced by caregivers of hospice care patients.”

#### 3.4.1 Synthesized finding 1: discomfort and changes experienced by caregivers of patients in hospice care

##### 3.4.1.1 Category 1: increased financial burden on the family

High cost of medical treatment: Most caregivers reported having to balance the patient’s medical expenses against daily living costs (“This has been a financial drain. We have no money now, but we still need to eat and put gas in the car.”) (20).

Work interruption: Caregivers’ employment is disrupted due to their caregiving responsibilities, resulting in severe financial hardship for the family (“Most of the time, I think to myself, I’m already deeply in debt, and I do not have time to work.”) (21).

##### 3.4.1.2 Category 2: a spectrum of complex emotions

Denial and avoidance: Faced with an incurable illness in their loved one, some caregivers initially responded with denial and avoidance (“I know she might die, but I just cannot believe it.”) (22).

Fear and anxiety: The diagnosis evoked intense fear and anxiety in caregivers (“When I first heard the diagnosis, I was absolutely terrified.”) (23) (“I wonder what might happen, but at the same time, I’m terrified. I’m afraid of losing him.”) (24).

Self-blame and helplessness: Many caregivers felt overwhelmed and helpless when confronted with the patient’s physical decline, often blaming themselves (“She’s weaker than before. We cannot manage her illness; there’s nothing we can do.”) (22), (“I feel powerless. I do not know how to help her.”) (20).

Resignation and acceptance: As the end of life approached, many caregivers shifted their focus from hoping for a cure to accepting the inevitability of death and preparing for it (“I’ve entrusted him to God. God will help us and take him peacefully.”) (22), (“I’ve come to accept that death is inevitable and try to take things one day at a time.”) (20).

##### 3.4.1.3 Category 3: disruption of daily life

Reduced social engagement: Both patients and caregivers experienced reduced social engagement due to the illness (“I worry that I’ll never get married because I spend so much time caring for her.”) (23), (“She does not really go out anymore. Seeing friends or family just stresses her out, makes her anxious and sad. “I cannot go out with friends to see a movie anymore because I have to take care of her. I do not do any of those things.”) (20).

Increased conflicts: Conflicts, both within the family and with others, intensified as the patient’s condition worsened and financial strain increased (“Sometimes, seeing him suffer so much, I wish he would just let go, but I cannot bear to lose him.”) (20), (“We do not have money, and she wants to give up treatment. We argue about it all the time.”) (22).

Sleep deprivation (“I wake up every two hours to give him his pain medication, then try to get back to sleep. It’s really hard.”) (21). (“It’s taking a toll on my body; I need more rest.”) (25).

Physical health decline: Caregivers often neglected their own health, leading to physical deterioration (“My father has been sick for over a year, in and out of the hospital. I’ve had headaches for years and take medicine for them. Now my back hurts badly too.”) (26).

#### 3.4.2 Synthesized finding 2: facilitating factors that enhance the comfort of family caregivers during hospice care

##### 3.4.2.1 Category 1: access to a professional hospice care team

Provision of professional caregiving guidance: Most caregivers expressed a need for professional guidance on caregiving skills and knowledge (“My mother’s swelling is getting worse, and she cannot move on her own. We need to prevent pressure ulcers, but seeing her like this, I just do not know what to do.”) (20).

Reduction of non-essential interventions: To enhance the comfort of end-of-life patients, certain non-essential procedures were discontinued. This not only reduced the burden on medical resources but also alleviated the financial and psychological strain on family caregivers (“Every time I see the machines and needles attached to him, it breaks my heart.”) (22).

##### 3.4.2.2 Category 2: humanistic and compassionate care

Empathetic communication from healthcare professionals (“I can communicate with the hospice doctors and nurses because they understand what I’m going through.” (25).

Genuine care and concern from the team (“This team truly cares about us. It’s like a good medicine or treatment in itself.”) (24).

Respect for and support of faith and spirituality (“Faith is such an important source of support. A deeply rooted belief is what keeps us going. My mother prays every night; she talks to God. I do the same. I think it really helps her and gives her a sense of security.”) (20).

##### 3.4.2.3 Category 3: a conducive care environment

Availability of professional medical equipment and support in the hospice care setting (“When you go home, you are left with your own devices—help and support are limited.”) (21).

A quiet and comfortable ward environment benefiting both patients and caregivers (“The hospital provides a safe, supportive environment. Once you are discharged and go home, you feel lonely and helpless.”) (27).

#### 3.4.3 Synthesized finding 3: family caregivers’ pursuit of multifaceted support

##### 3.4.3.1 Category 1: insufficient psychological and emotional support

Desire for regular psychological counseling services (“After the psychologist did their rounds last time, she also took me aside to talk with a counselor. I felt so much better. It would be great if we could have regular sessions.”) (26).

Need for emotional support from family and friends, which is often overlooked (“I think the hardest part is not knowing what state I’ll be in after work. Every day I’m exhausted from work. When he’s in a bad mood, I have to try to cheer him up.”) (20), (“My family does not pay attention to my needs or feelings. No one even calls to ask how I’m doing.”) (26).

##### 3.4.3.2 Category 2: insufficient social support

Desire for caregiver support groups to share experiences (“Here we can share our caregiving experiences with each other, and help and support one another.”) (26).

Regional disparities in hospice care quality (“We live in a rural area, far from the cancer center. Transportation is difficult, and the local medical care is very poor.”) (21).

Insufficient healthcare resources and overworked staff compromise patient care (“They’re very busy. They try to provide the best treatment, but they just do not have the time.”) (26).

Inadequacies in the medical insurance system: High treatment costs place an overwhelming burden on most families. Issues such as non-reimbursable medications, reduced coverage, and sudden policy changes leave families struggling to cope, highlighting an urgent need for national medical insurance reform (“Suddenly, the insurance is cutting back or changing things. Now we have to reconsider everything.”) (20).

Lack of financial and policy support (“Even though we set aside money for the illness and have some medical benefits, we are still struggling—trying to balance medical payments with housing costs, or just using up our savings to get by.”) (20), (“I tried to apply for help with the gas money for our daily trips to the cancer center, but it only covers three visits. You need receipts and the doctor’s signature, so I just gave up.”) (21).

## 4 Discussion

Through a review of the literature, we found that previous studies on the caregiving experience in palliative care primarily focused on the negative experiences and coping difficulties of caregivers. Although these studies summarized the challenges faced by caregivers, their perspective was largely limited to a problem-oriented approach, emphasizing the identification of caregivers’ vulnerabilities and difficulties (28–30). In contrast, this study adopts a positive, holistic theoretical perspective grounded in the concept of comfort. Using Kolcaba’s Theory of Holistic Comfort as the conceptual framework, it systematically synthesizes caregivers’ needs and experiences across four dimensions—physical, psychological and spiritual, sociocultural, and environmental—thereby shifting toward a more constructive and supportive model of caregiving.

### 4.1 Addressing caregivers’ comfort needs and providing psychological and emotional support

The synthesized findings revealed that caregivers of end-of-life patients face multiple care-related pressures, including economic, physical, and psychological challenges (21, 22, 27). Research indicates that caregivers become exhausted from witnessing their loved ones’ prolonged suffering while managing continuous caregiving responsibilities (31). Yang et al. (32) found that caregivers engaged in daily caregiving tasks yearn for opportunities to rest. Furthermore, Strom et al. (33) reported that the emotional support received by caregivers is often inadequate. Therefore, hospice care teams should prioritize caregivers’ comfort needs and provide them with multifaceted attention and support. As independent individuals, caregivers must balance their caregiving responsibilities with their own daily lives. Their personal sacrifices and caregiving capabilities should be acknowledged and supported, thereby enhancing their motivation to provide care, strengthening their sense of role identity and fulfillment, and ultimately achieving psychological and emotional comfort (34, 35). It is recommended that the comfort assessment of family caregivers be incorporated into the quality evaluation indicator system of hospice care, and that healthcare institutions be encouraged to establish a routine screening mechanism for caregivers’ needs.

### 4.2 Providing care guidance to caregivers and facilitating the establishment of caregiver alliances

Identifying and addressing the care needs of caregivers is crucial for enhancing the quality of hospice care. Early and continuous provision of professional knowledge and death education can help caregivers develop their caregiving competencies and emotional communication skills, enabling them to better cope with role conflicts and confront death with greater equanimity. The professional skills of the hospice care team should be improved, and regular training and assessments should be conducted. Stratified health education materials for family caregivers should be developed, including death education, caregiving skills training, and stress coping strategies, to help caregivers establish reasonable care expectations and self-protection awareness. Medical schools and clinical training institutions should be mobilized to promote the “family-centered” hospice care philosophy. Through simulation teaching and case discussions, the ability of interdisciplinary teams to support caregivers should be enhanced. Healthcare professionals should assist caregivers in establishing mutual support groups or peer education networks, utilizing both online and offline platforms to facilitate experience sharing and emotional expression. Through these platforms, professionals can also gain timely insights into caregivers’ challenges and needs, offering guidance and support to improve their comfort (36). Furthermore, it is recommended that patients and their caregivers be considered as a unified care unit, with multidisciplinary team collaboration involving clinical staff, social volunteers, psychologists, nutritionists, and community workers. Such collaboration enables comprehensive assessment of patient conditions and the development of tailored care plans, ultimately enhancing the comfort of both patients and their caregivers (37). Additionally, establishing a hospital-community-family tripartite linkage platform for continuity of care can optimize organizational structures and extend the high-quality nursing resources of tertiary hospitals to community-level facilities. This resource-sharing model, aimed at standardizing talent training and nursing consultations, can help supervise and ensure the quality of hospice care delivery (38). Finally, improving the professional competencies of hospice care teams, providing regular training and assessments for caregivers, establishing standards for ward environments and equipment, strengthening supervision and management, and developing a robust quality evaluation system are essential measures to enhance the comfort of both patients and their caregivers (39).

### 4.3 Improving the care environment and enhancing care convenience

Accumulating evidence suggests that the environment is a critical determinant of comfort for both patients and their caregivers (40, 41). Factors such as hospital bed quality, ward corridor space, and equipment layout have been identified as significant stimuli influencing comfort levels. Healthcare professionals should collaborate with hospital administrators to create a supportive and comfortable care environment for caregivers.

Research indicates that in many developed countries, most palliative care services are covered by medical insurance (23). However, in developing countries, the medical insurance system remains inadequate. The reimbursement rate for expensive medications is often low, placing a substantial financial burden on ordinary families who struggle to afford high medical costs. Therefore, it is imperative that medical insurance authorities prioritize the establishment of a rational drug reimbursement system, simplify reimbursement procedures, and alleviate both the economic and caregiving burdens on families.

The synthesized findings also reveal significant disparities in hospice care development across different regions (23). In many developing countries, hospice care remains largely theoretical, with only a limited number of hospitals equipped with specialized hospice wards and professional teams. To address this gap, it is recommended to establish a multicenter hospital network that facilitates resource sharing, enables continuous monitoring of patient conditions and treatment trajectories, improves the quality and accessibility of medical services, and ultimately provides greater care convenience for both patients and their caregivers.

### 4.4 Mobilizing community and social support to alleviate caregiving burden

Economic pressure is a primary concern for most patient families. Healthcare professionals can assist charitable organizations in establishing hospice care financial assistance programs to alleviate the economic burden on disadvantaged families. Canada has introduced the Compassionate Care Benefit (CCB) to help employed individuals balance work and end-of-life caregiving responsibilities, preventing caregivers from facing financial collapse due to leaving their jobs while also protecting their right to return to their original positions (42). Meanwhile, some countries (e.g., Belgium) offer paid palliative care leave (43). It is recommended to explore the development of economic assistance policies for caregivers, such as caregiving allowances, tax reductions, and the inclusion of family caregivers under long-term care insurance coverage, in order to alleviate their financial burden. Research indicates that hospice volunteer services can alleviate caregiving burdens and reduce healthcare costs (44). It is recommended that medical students and social workers be mobilized to participate in hospice care volunteering, and that a hospice care volunteer alliance be established to provide compassionate services for end-of-life patients, including basic hygiene care and psychological support. Government agencies and hospitals should jointly develop a volunteer training system to standardize hospice care volunteer services. On the other hand, community hospice care centers could be expanded to reduce medical costs, conserve healthcare resources, and alleviate the caregiving burden on family caregivers.

## 5 Conclusion

This study synthesized the comfort experiences of family caregivers in hospice care, providing an in-depth understanding of caregivers’ needs and comprehensively analyzing the factors influencing comfort in hospice care. The main problems identified include caregiver role conflict, high financial burden, and insufficient social support. The findings suggest that healthcare professionals should pay attention to caregivers’ multidimensional comfort needs. It is recommended that the comfort assessment of family caregivers be incorporated into the quality evaluation indicator system of hospice care, and that healthcare institutions be encouraged to establish a routine screening mechanism for caregivers’ needs. Caregivers should be provided with care guidance to help them establish reasonable care expectations and self-protection awareness. Governments, healthcare institutions, and non-governmental organizations should be mobilized to provide economic support and institutional safeguards, such as caregiving allowances and tax reductions, to alleviate financial burden, thereby providing evidence-based support for future research.

Although this study attempted to cover relevant literature through systematic searches of international and Chinese databases, the number of studies that met the inclusion criteria was relatively small. This may limit the generalizability of the findings to some extent. Moreover, studies published in other languages and gray literature were not included, introducing language and publication bias. Furthermore, although this study graded the credibility of individual findings using the JBI criteria, it did not perform an overall credibility rating of the synthesized findings, which may limit the systematic assessment of the strength of evidence. Future research could further validate and expand upon the conclusions of this study by conducting larger-scale empirical studies or mixed-methods research, and could also consider applying ConQual or other credibility assessment tools to further validate the reliability of the conclusions drawn.

This study primarily used Kolcaba’s Theory of Comfort to organize and interpret the synthesized findings. Although this theory is a mainstream framework in the field of comfort research, other potential theoretical perspectives (e.g., stress-coping theory) were not systematically incorporated into the analysis, which may limit the depth of multi-theoretical interpretation of caregivers’ comfort experiences. Future research could adopt a multi-theoretical integration framework or compare the similarities and differences of synthesized findings under different theoretical perspectives. Moreover, there are considerable differences in healthcare systems, hospice care service models, and cultural backgrounds across different countries and regions. These differences may influence caregivers’ experiences, needs, and their understanding of comfort. Therefore, caution should be exercised when applying the conclusions of this study, taking into account the specific sociocultural and institutional contexts.

## Data Availability

The original contributions presented in the study are included in the article/supplementary material, further inquiries can be directed to the corresponding author.

## References

[1] Bray F Laversanne M Sung H Ferlay J Siegel RL Soerjomataram I . Global cancer statistics 2022: GLOBOCAN estimates of incidence and mortality worldwide for 36 cancers in 185 countries. CA Cancer J Clin. (2024) 74:229–63. doi: 10.3322/caac.21834, 38572751

[2] Ketcher D Trettevik R Vadaparampil ST Heyman RE Ellington L Reblin M. Caring for a spouse with advanced cancer: similarities and differences for male and female caregivers. J Behav Med. (2020) 43:817–28. doi: 10.1007/s10865-019-00128-y, 31845168 PMC8936415

[3] Wang L Li Y Zhao R Li J Gong X Li H. Influencing factors of home hospice care needs of family caregivers of the older adult with chronic diseases at the end of life in China: a cross-sectional study. Front Public Health. (2024) 12:1348285. doi: 10.3389/fpubh.2024.1348285, 38756894 PMC11098011

[4] Deng Y Wan H Liu X Zhao Z Wang X. Meta-synthesis of qualitative research on family caregiving experiences for individuals with cognitive impairment: a systematic review. Front Public Health. (2025) 13:1701561. doi: 10.17605/OSF.IO/3CZ26, 41573790 PMC12819226

[5] Kolcaba KY Fisher EM. A holistic perspective on comfort care as an advance directive. Crit Care Nurs Q. (1996) 18:66–76. 8689455 10.1097/00002727-199602000-00009

[6] Kirby E McLaughlan R Bellemore F Swanson R Gissing J Chye R. On comfort in palliative care. Health Sociol Rev. (2025) 34:25–41. doi: 10.1080/14461242.2024.2447021, 39882612

[7] Cole L Collins T Speyer R Ellis C Cordier R. Exploring the lived experience of loneliness and social isolation in informal palliative caregivers: a systematic review. Palliative Care Soc Prac. (2025) 19:1–15. doi: 10.1177/26323524251397000 PMC12681630 41362639

[8] Cole L Collins T Speyer R Ellis C Cordier R. Exploring the lived experience of loneliness and social isolation in informal palliative caregivers: a systematic review. Palliative Care Soc Prac. (2025) 19:26323524251397000. doi: 10.1177/26323524251397000 PMC12681630 41362639

[9] Patano A Wyatt G Lehto R. Palliative and end-of-life family caregiving in rural areas: a scoping review of social determinants of health and emotional well-being. J Palliat Med. (2024) 27:1229–46. doi: 10.1089/jpm.2023.0566, 38598274

[10] Luth EA Maciejewski PK Phongtankuel V Xu J Prigerson HG. Associations between hospice care and scary family caregiver experiences. J Pain Symptom Manag. (2021) 61:909–16. doi: 10.1016/j.jpainsymman.2020.08.041, 33038426 PMC8024420

[11] Wen F Hsieh C Chou W Chen J Chang W Tang S. Associations of Taiwanese patient-caregiver concordance on death preparedness with dyadic end-of-life outcomes. SSM-Mental Health. (2025) 8:100553. doi: 10.1016/j.ssmmh.2025.100553

[12] Loscalzo MJ. Palliative care: an historical perspective. Hematology Am Soc Hematol Educ Program. (2008) 2008:465. doi: 10.1182/asheducation-2008.1.465 19074127

[13] Kolcaba KY Kolcaba RJ. An analysis of the concept of comfort. J Adv Nurs. (1991) 16:1301–10. 1753026 10.1111/j.1365-2648.1991.tb01558.x

[14] Foley G. The supportive relationship between palliative patients and family caregivers. BMJ Support Palliat Care. (2018) 8:184–6. doi: 10.1136/bmjspcare-2017-001463 29353254

[15] Stajduhar KI Nickel DD Martin WL Funk L. Situated/being situated: client and co-worker roles of family caregivers in hospice palliative care. Soc Sci Med. (2008) 67:1789–97. doi: 10.1016/j.socscimed.2008.09.012, 18922609

[16] Lin J He Z Fan G. Determinants of quality of life in primary family caregivers of patients with advanced cancer: a comparative study in southern China. Front Public Health. (2023) 11:1034596. doi: 10.3389/fpubh.2023.1034596, 37304122 PMC10248401

[17] Son H-M. A literature review for qualitative Meta-synthesis. Korean Association Qualitative Res. (2020) 5:109–18. doi: 10.48000/KAQRKR.2020.5.109

[18] Lockwood C Munn Z Porritt K. Qualitative research synthesis: methodological guidance for systematic reviewers utilizing meta-aggregation. JBI Evidence Implementation. (2015) 13:179–87. doi: 10.1097/XEB.0000000000000062, 26262565

[19] Treloar C Champness S Simpson PL Higginbotham N. Critical appraisal checklist for qualitative research studies. Indian J Pediatr. (2000) 67:347–51. 10885207 10.1007/BF02820685

[20] Ferrell BR Kravitz K Borneman T Taratoot FE. Family caregivers: a qualitative study to better understand the quality-of-life concerns and needs of this population. Clin J Oncol Nurs. (2018) 22:286–94. doi: 10.1188/18.CJON.286-294, 29781459

[21] Washington KT Benson JJ Chakurian DE Popejoy LL Demiris G Rolbiecki AJ . Comfort needs of cancer family caregivers in outpatient palliative care. J Hosp Palliat Nurs. (2021) 23:221–8. doi: 10.1097/njh.0000000000000744 33605647 PMC8084891

[22] Benites AC Rodin G de Oliveira-Cardoso ÉA Dos Santos MA. "you begin to give more value in life, in minutes, in seconds": spiritual and existential experiences of family caregivers of patients with advanced cancer receiving end-of-life care in Brazil. Support Care Cancer. (2022) 30:2631–8. doi: 10.1007/s00520-021-06712-w, 34817692 PMC8611251

[23] Twahirwa JC Mukeshimana M Fitch M Katende G. Palliative care conditions managed at home and self-reported challenges experienced by informal home-based caregivers in Rwanda: a qualitative study. BMC Palliat Care. (2025) 24:267. doi: 10.1186/s12904-025-01905-0, 41131522 PMC12548109

[24] Jin L Zhang Q. Qualitative study in nursing experience of spouses taking care of patients in terminal stage of cancer in family-like ward. Mil Nurs. (2016) 33:20–23+47.

[25] Leow MQH Chan SWC. The challenges, emotions, coping, and gains of family caregivers caring for patients with advanced cancer in Singapore: a qualitative study. Cancer Nurs. (2017) 40:22–30. doi: 10.1097/NCC.0000000000000354, 26925989

[26] Wu H Zhou N Chen X Liang G. Qualitative study of caregiver burden and needs in hospice care patients with advance cancer. Chinese Medical Ethics. (2019) 32:1566–70.

[27] Perão OF Nascimento ERP Padilha MICS Lazzari DD Hermida PMV Kersten MAC. Social representations of comfort for patients’ family members in palliative care in intensive care. Rev Gaucha Enferm. (2021) 42. doi: 10.1590/1983-1447.2021.20190434, 33656163

[28] Li Y Shi S Lu Y Zhang D Liu R Chen Y . The burden and needs of primary caregivers in home-based palliative care: a qualitative study based on social-ecological systems theory. BMC Palliat Care. (2026) 25:61. doi: 10.1186/s12904-026-02001-7, 41652369 PMC12973859

[29] Nicoli F Grossi A Picozzi M. Caregivers and family members’ vulnerability in end-of-life decision-making: an assessment of how vulnerability shapes clinical choices and the contribution of cclinical ethics consultation. Philosophies. (2024) 9:14. doi: 10.3390/philosophies9010014

[30] Sood A Shruti Sharma S Kaur Arora M. Voices of resilience: lived realities and challenges of caregivers for cancer patients receiving palliative care – a qualitative perspective. Indian J Palliat Care (2026) 32:78–84. doi: 10.25259/IJPC_265_2025 41953833 PMC13054368

[31] Rezaei M Keyvanloo Shahrestanaki S Mohammadzadeh R Aghili MS Rajabi M. Caregiving consequences in cancer family caregivers: a narrative review of qualitative studies. Front Public Health. (2024) 12:1334842. doi: 10.3389/fpubh.2024.1334842 38584929 PMC10997218

[32] Yang Y Liang X Liu Q Liu J. Navigating limited resources: experiences of caregivers for elderly terminal cancer patients in a region with limited palliative care services. Support Care Cancer. (2025) 33:207. doi: 10.1007/s00520-025-09270-7, 39971822

[33] Strøm A Andersen KL Korneliussen K Fagermoen MS. Being "on the alert" and "a forced volunteer": a qualitative study of the invisible care provided by the next of kin of patients with chronic heart failure. J Multidiscip Healthc. (2015) 8:271–7. doi: 10.2147/JMDH.S82239, 26082643 PMC4461135

[34] Alam S Hannon B Zimmermann C. Palliative care for family caregivers. J Clin Oncol. (2020) 38:926–36. doi: 10.1200/jco.19.00018 32023152

[35] Uzun U Başar S Saritaş A. Spiritual needs of family caregivers in palliative care. BMC Palliat Care. (2024) 23:256. doi: 10.1186/s12904-024-01589-y, 39511622 PMC11542245

[36] Nysaeter TM Olsson C Sandsdalen T Hov R Larsson M. Family caregivers' preferences for support when caring for a family member with cancer in late palliative phase who wish to die at home - a grounded theory study. BMC Palliat Care. (2024) 23:15. doi: 10.1186/s12904-024-01350-5, 38212707 PMC10782637

[37] De Vleminck A Matthys O Turola E Dierickx S Dombrecht L Van Goethem V . Impact of a nurse-led and a web-based psychoeducational program for advanced cancer patients and their caregivers: results of a three-arm randomized controlled trial. Int J Nurs Stud. (2025) 171:105192. doi: 10.1016/j.ijnurstu.2025.105192, 40925214

[38] Mori M Morita T. Advances in hospice and palliative care in Japan: a review paper. Korean J Hosp Palliat Care. (2016) 19:283–91. doi: 10.14475/kjhpc.2016.19.4.283

[39] Bauman C Årestedt K Wallin V Tibell LH Fürst P Hudson P . Web-based psychoeducational intervention to improve family caregiver preparedness in specialized palliative home care: a randomized controlled trial. Psychooncology. (2025) 34:e70202. doi: 10.1002/pon.70202, 40485035 PMC12146470

[40] Teo KY Yuan LX Kan SH Ho AL Ng GNJ. Transforming caregivers into partners: advancing WHO patient safety goals in Singapore acute hospital. BMJ Open Qual. (2025) 14:e003555. doi: 10.1136/bmjoq-2025-003555, 41326054 PMC12684129

[41] O’Hara S Knill K Courville K Kiumarsi A Hazra S Cheng Z . Rapid review of the health care built environment support for hospice/end-oflife patients, families, and interdisciplinary care teams. J Hosp Palliat Nurs. (2026) 28:15–23. doi: 10.1097/NJH.0000000000001187, 41400387

[42] Gardiner C Stajduhar K. Internationally transferable policy solutions for supporting employed end of life family caregivers: Canadian compassionate care benefit. Prog Palliat Care. (2025) 33:166–77. doi: 10.1080/09699260.2025.2524231

[43] Maetens A Beernaert K Deliens L Aubry R Radbruch L Cohen J. Policy measures to support palliative care at home: a cross-country case comparison in three European countries. J Pain Symptom Manag. (2017) 54:523–529.e5. doi: 10.1016/j.jpainsymman.2017.07.022, 28736105

[44] Nassehi A Saake I Breitsameter C Bauer A Barth N Reis I. Adding spontaneity to organizations - what hospice volunteers contribute to everyday life in German inpatient hospice and palliative care units: a qualitative study. BMC Palliat Care. (2024) 23:81. doi: 10.1186/s12904-024-01409-3, 38539136 PMC10976705

